# Review of the nutritional benefits and risks related to intense sweeteners

**DOI:** 10.1186/s13690-015-0092-x

**Published:** 2015-10-01

**Authors:** Olivier Bruyère, Serge H. Ahmed, Catherine Atlan, Jacques Belegaud, Murielle Bortolotti, Marie-Chantal Canivenc-Lavier, Sybil Charrière, Jean-Philippe Girardet, Sabine Houdart, Esther Kalonji, Perrine Nadaud, Fabienne Rajas, Gérard Slama, Irène Margaritis

**Affiliations:** 1 Department of Public Health, Epidemiology and Health Economics, University of Liege, CHU Sart Tilman, Bât B23, 4000, Liège, Belgium; 2 CNRS UMR 5293/Université de Bordeaux, Bordeaux, France; 3 Centre Hospitalier de Luxembourg, Luxembourg, Luxembourg; 4 Université de Picardie, Amiens, France; 5 Centre Hospitalier Universitaire Vaudois, Lausanne, Suisse Switzerland; 6 Centre des Sciences du Goût et de l’Alimentation - INRA Dijon, Dijon, France; 7 Université Claude Bernard Lyon 1, Hospices Civils de Lyon, Inserm U1060, Lyon, France; 8 Sorbonne Universités, UPMC Univ Paris 06, Paris, France; 9 French Agency for Food, Environmental and Occupational Health & Safety (Anses), Maisons-Alfort, France; 10 INSERM 855/Université Claude Bernard Lyon 1, Lyon, France; 11 Hôtel-Dieu Hospital, René Descartes University-Paris V, Paris, France

**Keywords:** Intense sweetener, Aspartame, Acesulfame K, Stevia, Sucralose, Diabetes, Obesity, Energy intakes, Satiety, Eating behaviour, Compensation, Weight

## Abstract

**Background:**

The intense sweeteners currently authorised in Europe comprise ten compounds of various chemical natures. Their overall use has sharply risen in the last 20 years. These compounds are mainly used to formulate reduced-calorie products while maintaining sweetness.

**Methods:**

This extensive analysis of the literature reviews the data currently available on the potential nutritional benefits and risks related to the consumption of products containing intense sweeteners.

**Results and Conclusions:**

Regarding nutritional benefits, the available studies, while numerous, do not provide proof that the consumption of artificial sweeteners as sugar substitutes is beneficial in terms of weight management, blood glucose regulation in diabetic subjects or the incidence of type 2 diabetes. Regarding nutritional risks (incidence of type 2 diabetes, habituation to sweetness in adults, cancers, etc.), it is not possible based on the available data to establish a link between the occurrence of these risks and the consumption of artificial sweeteners. However, some studies underline the need to improve knowledge of the links between intense sweeteners consumption and certain risks.

## Background

The term 'Intense sweeteners' (IS) refers to various substances of plant origin or obtained by chemical synthesis, used in the food industry for their high sweetening power and their low caloric value. The intense sweeteners currently authorised in Europe comprise ten compounds of various chemical natures.

They are used in the formulation of foods and beverages, essentially for their sweetening role but also for their technological properties (stabilisers, texturisers). Their sweetening power is a hundred (e.g. acesulfame K, aspartame) to several thousand (e.g. neotame) times higher than that of sucrose. Their overall use has sharply risen in the last 20 years. These compounds are mainly used to formulate reduced-calorie products while maintaining sweetness. Their lower calorie content suggests a direct link to weight management for consumers. However, no claim- related to the effects of IS on weight management have been authorised.

This extensive analysis of the literature reviews the data currently available on the potential nutritional benefits and risks related to the consumption of products containing IS, particularly since nutritional benefits were not examined in the context of the initial authorisation issued under Regulation (EU) No 257/2010.

## Methods

The substances covered in this assessment are the IS currently authorised in Europe, after scientific review: aspartame [1], acesulfame potassium (K) [2], cyclamic acid and its salts [3], steviol glycosides [4], neohesperidin dihydrochalcone [5], neotame [6], saccharine and its salts [7], sucralose [8], aspartame-acesulfame salt [2] and thaumatin [5].

The search strategy aimed to find both published and unpublished studies. A three-step search strategy was utilised in this review. An initial search of Medline (Table 1), Cochrane Database of Systematic Reviews and Psychinfo was undertaken, followed by analysis of the text words contained in the title and abstract, and of the index terms used to describe the article. A second search using all identified keywords and index terms diet was then undertaken across all included databases. Thirdly, the reference list of all identified articles was searched for additional studies. No language restriction was applied. Moreover, food industries and consumer associations were contacted to know if they were aware of unpublished studies.

**Table 1:** Search strategy in Medline

1. Sweetening agent*.mp
2. Sweetener*.mp
3. Sweetened.mp
4. Aspartam*.mp
5. Aspartam*-acesulfame salt*.mp
6. Acesulfam*.mp
7. Acesulfame potatium.mp
8. Acesulphame.mp
9. Cyclamat*.mp
10. Stevia*.mp
11. Steviol*.mp
12. Neohesperidin dihydrochalcone.mp
13. Neotam*.mp
14. Saccharin*.mp
15. Sucralose*.mp
16. Thaumatin*.mp
17. Low calori*.mp
18. Sugar substitue*.mp
19. Diet* drink*.mp
20. Diet* refreshement*.mp
21. Diet* food*.mp
22. Diet* chocolat*.mp
23. Diet* yoghourt*.mp
24. Diet* milk*.mp
25. Diet* beverage*.mp
26. Diet* jam*.mp
27. Diet* marmalade*.mp
28. Diet* candy*.mp
29 Diet* cookie*.mp
30. Diet* biscuit*.mp
31. Diet* cracker*.mp
32. Diet* eat*.mp
33. Diet* cuisine*.mp
34. Diet* cook*mp
35. Diet* meat.mp
36. Diet* nutriment*.mp
37. Diet* nutrition*.mp
38. Diet*menu*.mp
39. Diet* dish*.mp
40. Diet* drinkable*.mp
41. Diet* drinking*.mp
42. Diet* juice*.mp
43. Or/1-16
44. Or/17-42
45. Or/43-44
46. Limit 45 to humans

Types of studies were broad and unrestrictive to capture as much data as possible. Meta-analysis, randomised controlled, quasi experimental, cohort, case-control and cross-sectional studies were accepted. *In vitro*, *ex vivo*, and animals studies have been excluded.

Whatever its design and quality, a single study is never able to establish the causality between the exposure and the disease, in this case between IS consumption and its effects on health. In our review, we used part of Hill’s criteria of causation to assist in the assessment of the causal relationships. Indeed, a modified version of the Bradford Hill criteria was used to evaluate the evidence of a causal relationship between IS consumption and health outcomes. As a matter of fact, the following eight criteria, considered as the most important to answer our questions, were used in our review: strength, consistency, temporality, coherence, experiment, plausibility, analogy and biological gradient. One criterion, the specificity, was omitted because considered as nonspecific to our various outcomes. It should be pointed that none of these criteria alone is sufficient to establish causality and that no systematic algorithm was used. The final interpretation of the causality was based on experts’ judgment based on all analysed data.

## Results

The exhaustive review included 10,989 manuscripts (9,965 in English and 1024 in non-English languages). Out of them, 9,373 were excluded after a reading of the title and the abstract. Consequently, 1,616 full-text manuscripts have been extensively reviewed of which 383 (all in English) have been considered of interest for the topic of benefits and risks related to IS. For this review however, only the most relevant ones have been quoted, mainly based on their methodology, novelty and originality. Among these studies, 30.1 % were funded by the industry, 56.3 % by non-profit organisations, and the others did not report funding sources. However, we did not consider differently studies based the funding source.

### Effects on eating behaviour and taste preferences

IS are often consumed as sugar substitutes, particularly in beverages, in order to satisfy a desire for sweetness while avoiding energy intake from sugars. The expert appraisal assessed whether there were metabolic consequences of this separation of sweetness and calorie intake, particularly in terms of body's ability to associate a taste with an energy value and therefore regulate its energy balance, and also in terms of consequences of IS consumption on appetite for sweetness and the consumption of sweet products.

#### Data in adults

A meta-analysis covering studies undertaken before 2006 along with around ten randomised experimental studies were identified to address these points. The meta-analysis of 15 randomised experimental studies [9] assessed the effects of aspartame consumed alone or with other (unspecified) IS on food and energy intake during the course of a day in adults. These measurements covered a limited number of subjects (less than 30) and highly variable time periods of a few days to 16 weeks. The main inclusion criterion for studies in this meta-analysis was the measurement of food intakes for at least 24 hours, to assess the full extent of any compensatory effects of the various meals consumed over the day. The authors conclude that consuming aspartame as a sugar substitute results in a decrease of daily energy intake by 220 Kcal on average. Moreover, the authors indicate that this substitution may be more efficient in beverages than in solid foods, since the energy supplied by liquids leads to less satiety than that supplied by solid foods. In fact, the estimated compensation rate is thought to be lower for sugars consumed in liquid form than in solid form [10]. Therefore, according to these authors, the reduction in energy intake due to the replacement of sugar with sweeteners is greater with artificially sweetened beverages than with artificially sweetened solid foods. However, the conclusions of this meta-analysis should be treated with caution, due to several methodological limitations, particularly a lack of essential information on the study selection process, the assessment of their quality and the statistics applied to assess the heterogeneity of the data taken into account. Other experimental studies (that were not included in the meta-analysis since they covered periods of less than 24 hours) analysed the effects of IS on appetite and food intake. These studies used an IS preload approximately one hour before a meal, generally in beverage form (rarely in solid form, i.e. in a food), and measured food intake and calorie intake during the next meal. All of these studies showed that irrespective of the nature of the tested IS, a preload reduced the sensation of hunger and the desire to eat, with a maximum effect immediately after its consumption. However, this effect tended to disappear before the start of the meal, which explains why most studies did not observe reduced food intake during the meal after the preload. Regarding food preferences, several studies assessed the effect of IS on the perception of sweetness (gustatory stimuli) and/or taste preferences for foods. Several studies showed that preference for a sweet food was independent of the sweetening agent (i.e. no difference between an IS and sucrose), but their results differed as to the repercussions of this preference on consumption of this food. However, these studies had extremely variable protocols and objectives, to the extent that it is difficult to compare their results and draw an overall conclusion on the effect of IS on food preferences.

Overall, based on studies dealing with occasional exposure to an IS before a meal, it is not possible to infer the effect of regular IS consumption on sweetness habituation or increased cravings for sweetened products. Most experimental studies show that the occasional consumption of IS before or during a meal has no effect on food intake or energy intake during the next meal. Occasional IS consumption before a meal reduces the sensation of hunger and the desire to eat, just like caloric sweeteners, but this effect is temporary and disappears before the start of the meal. In most cases, the use of IS as sugar substitutes results in a decrease in short-term energy intake due to their low calorie content and the lack of compensation. However, the available data cover insufficient time periods to guarantee the maintenance of this effect over the medium or long term.

#### Data in children

Preference for sweetness is innate. It is strong at birth and then tends to decrease. However, it seems to be maintained by the repeated consumption of sweetened foods or beverages during early childhood [11]. A study [12] showed that adding aspartame or sucrose to milk favoured its consumption. Moreover, the work of Birch and their collaborators revealed that children preferred flavours associated with calorie intake, suggesting that sweetness itself is not sufficient to generate food preferences, and that energy density, just as much as (or even more than) sweetness, can determine food preferences [13, 14]. However, there are no data showing whether IS have a specific effect, in relation to caloric sweeteners, on the development of taste and food preferences. A study compared the effects of consuming 250 mL daily of artificially sweetened beverages *vs* sugar-sweetened beverages on the satiety and desire to eat of children aged seven to 11 years for 18 months [15]. The level of satiety was the same, irrespective of the beverage consumed.

In conclusion, based on the available studies, it is not possible to determine whether IS consumed during early childhood have a specific effect on the development of taste and food preferences or on the short- and medium-term regulation of food intake.

### Effects on body weight and composition

IS are commonly used by consumers as sugar substitutes as part of weight-loss diets or to control energy intake and prevent weight gain.

#### Data in adults

Two meta-analysis (Table 2) [9, 16], a systematic review [17] and several original articles [18–29]examined the relationship between IS consumption and changes in body composition and weight. A meta-analysis [30] took into account observational studies (with nine articles included) and randomised controlled trials (RCTs, with 15 articles included) in adults and children. The section on observational studies showed no relationship between IS consumption and changes in body weight or fat mass but showed a slight increase in BMI (+0.03 kg/m^2^ on average). The section on RCTs showed that replacing sugars with IS in sweet products resulted in moderate weight loss (with an estimated average effect of 0.8 kg) and a decrease in BMI (-0.24 kg/m^2^ on average) for time periods ranging from three weeks to 18 months. This meta-analysis, of good methodological quality, highlights the extreme variability of results from studies with a similar design (whether RCT or observational) and the differences in results between observational studies and RCTs. In the meta-analysis by De la Hunty [9], eight studies on very heterogeneous populations (people with energy restrictions and unrestricted diets, normal-weight and obese people, in normal living conditions and in metabolic chambers) were included. According to the authors, the effect of IS on weight loss is significant. They extrapolate the theoretical reduction of 220 kcal/day related to the replacement of sugars with aspartame over the long term, and by postulating its maintenance over time, calculate that this reduction could result in weight loss of 0.2 kg per week. However, the methodological weaknesses of this meta-analysis has to be emphasised, particularly the lack of essential information related to the study selection process and the statistics applied to assess heterogeneity. The systematic review by Wiebe et al. (2011) cites two intervention studies comparing the effects of artificially sweetened drinks and sugar-sweetened drinks on BMI [31,32]. These studies, focusing on different populations (normal-weight women in one and overweight women in the other), had different results (no effect in normal-weight women, reduced weight in overweight women). Five other randomised controlled trials [21, 24, 25, 27, 29] were identified. They were all undertaken in overweight subjects and the majority focused on very small populations (between 20 and 50 subjects). Two demonstrated modest weight loss of 1.2 and 1.5 kg on average, but the other three, including the one with the largest study population (*n* = 318), did not show any effects on weight loss related to the consumption of artificially sweetened beverages compared to the consumption of sugar-sweetened beverages or water. There are also seven prospective observational epidemiological studies with highly heterogeneous results. One study did not show any association between IS consumption and changes in body composition [23]; four studies reported a positive association, i.e. a significantly higher body weight or waist size in IS consumers [18–20,28]; and two studies reported a negative association [22, 26].

**Table 2:** methodology of the meta-analyses on the effect of intense sweeteners on body composition.

First Author, year of publication (reference)	Search date	Database used	Population	Study design	Main findings	Heterogeneity	Publication bias
de la Hunty, 2006 (ref 9)	Not clear	Not clear	Healthy adults	RCT (N = 9)	Non-significant effect size of 0.221 (0.000 to 0.443) standard deviation of weight loss	Not assessed	Not assessed
Miller, 2014 (ref 16)	16 September 2013	PubMed + manual search	Healthy adults and children	RCT (N = 15) Prospective study (N = 9)	1. RCT:		No evidence of publication bias (Egger’s regression test)
					- on weight: -0.80 kg (–1.17 to –0.43)	I^2^ 61 %	
					- on body mass index: –0.24 kg/m^2^ (–0.41 to –0.07)	I^2^ 0 %	
					2. Prospective studies		
					- on weight: 0.02 kg (–0.01 to –0.06)	I^2^ 92 %	
					- on body mass index: 0.03 kg/m^2^ (0.01 to 0.06)	I^2^ 53 %	

In conclusion, observational and intervention studies report contradictory associations between IS consumption and weight loss. Therefore, no conclusion can be drawn as to the long-term effect of replacing caloric sweeteners with IS on the weight of regular adult consumers of sweet products.

#### Data in children

Four RCT studies focusing on the relationship between IS consumption and body composition were identified. In three of these studies, changes in weight and BMI did not differ between IS consumers and non-consumers [33–35]. These studies focused on overweight or obese children and had methodological limitations. The fourth study, of good methodological quality, examined the effects of consuming 250 mL/day of an artificially sweetened drink, compared to the same amount of sugar-sweetened drink, in 641 normal-weight children (aged four to 12 years), who were regular consumers of sugar-sweetened drinks, for 18 months [36]. This study showed a significant decrease in the BMI z-score (the most relevant criterion to assess changes in corpulence in growing children) in the group that consumed artificially sweetened drinks. The change in body weight between the two groups differed by 1 kg on average. Of the seven prospective epidemiological studies in children, five [37, 38] observed a positive relationship between IS consumption (primarily in beverage form) and weight over time, while two [37] did not find any relationship. To explain these findings, the authors of these studies assumed that subjects 'at risk for weight gain' or with less healthy food profiles were those who consumed the most IS in order to reduce their energy intake.

Most of the prospective observational studies undertaken in children show that IS use is paradoxically associated with weight gain, although the causality of this relationship has not been established. The four available controlled trials showed conflicting results but none reported weight gain. No conclusions can be drawn from all of these studies as to the significance of IS for weight management in children and adolescents.

### Effects on blood glucose and type 2 diabetes

This section presents data on the effects of IS consumption on glucose homeostasis and risk of diabetes, in healthy subjects, type 1 diabetics and type 2 diabetics Thirty-one clinical trials and two reviews assessed the short-term effects (less than one week) of IS consumption on glucose homeostasis. To date, the data on the long-term risk of developing diabetes are still limited and have been taken from seven observational epidemiological studies.

#### Effects on glucose homeostasis

Regarding the acute effects (i.e. less than 24 hrs.), the available studies did not show any effects related to the consumption of aspartame on an empty stomach [39–44], saccharine [45] or sucralose [46, 47] on blood glucose and insulin levels. Other studies assessed acute effects of IS on post-prandial glycaemic parameters after a test meal [48–52]. These studies generally showed that consuming IS before a test meal did not modify post-prandial glycaemic and insulin responses compared to a placebo, and reduced these responses compared to a sucrose preload. These effects were reported irrespective of the tested IS (aspartame, stevia extract, sucralose, beverage containing acesulfame K and sucralose). It should also be noted that the parameters of these studies were highly variable with differences in the composition of test meals, the time between the preload and the meal, the studied subject groups (age, sex, healthy overweight or obese subjects) and the preload form (solid or liquid). Several studies also showed that consuming IS before a meal resulted in increased secretion of GLP1 (Glucagon-like peptide), a gastro-intestinal hormone that usually increases insulin secretion, slows down gastric emptying and reduces glucagon secretion [53]. This increase in GLP1 may be induced by IS activating sweetness receptors, as suggested by data in rats [54].

When considering short- and medium-term effects, several studies assessed the effect of regular IS intake (one to three times per day, for a few days to several weeks), in capsule form or in beverages, on maintaining blood sugar control (glucose and insulin concentrations measured after a night of fasting, glycated haemoglobin HbA1c). For type 2 diabetics, the consumption of sucralose [55] or aspartame [56–58] for periods of up to 18 weeks did not change fasting glucose levels) compared to sucrose or a placebo. Furthermore, fasting plasma glucose and insulin, and HOMA-IR (insulin sensitivity calculated from the HOMA index) were not modified in non-diabetic obese subjects who had consumed a beverage sweetened with aspartame for six months compared to groups who had consumed sugar-sweetened drinks, water or milk [21]. Other studies covering unspecified IS compared to sucrose in obese or overweight subjects [59] confirm these results. Regarding stevia extracts, the data show either a lack of effect on glucose control in healthy [59] or diabetic [59] subjects or a slight significant decrease in blood glucose levels in healthy subjects [59] or hypertensive subjects [60].

Overall, the vast majority of studies do not show any acute effects of IS intake on blood glucose or insulin concentrations measured on an empty stomach or after a test meal, in healthy subjects or in diabetics. Some studies reported a modest increase in GLP-1 secretion, but with no repercussions on insulin secretion or blood glucose concentrations. IS consumption has no effect on short- and medium-term blood glucose parameters in healthy subjects or in diabetics.

#### Effects on the risk of type 2 diabetes (T2D)

The seven observational studies dealing with IS consumption and the incidence of T2D showed diverging results. Four cohort studies of good quality (three undertaken in North American populations and one in a European population), over periods of nine to 24 years, did not show any relationship between the consumption of artificially sweetened beverages and the risk of developing T2D after adjustment for BMI and energy intake of subjects [61–63, 26]. Three other cohort studies suggested a positive association between the consumption of artificially sweetened beverages and the incidence of T2D [64–66]. Among them a French study, [64], showed that the incidence of T2D was significantly higher (HR (95 % CI) 2.21 [1.56-3.14]) in the group of women consuming the largest amounts of artificially sweetened beverages (over 600 mL per week) who were monitored for 14 years, with a linear and dose-dependent relationship. The second study, undertaken in the United States for seven years, reported an increase in the incidence of T2D in subjects consuming more than one artificially sweetened beverage per day in a model with adjustment for the primary confounding factors (HR (95 % CI) 1.67 [1.27–2.20]. The third study, which reported an increased incidence of T2D in subjects consuming more than one artificially sweetened beverage per week (HR (95 % CI) 1.70 [1.13–2.55], focused on a limited-sized Japanese population not representative of the general population, monitored for seven years. It is important to underline the heterogeneity of these data, particularly in terms of the characteristics of the populations and the monitoring periods (from seven to 24 years). Furthermore, in these studies, the consumption of artificially sweetened beverages was recorded when the subjects were first included, often through self-administered frequency questionnaires, with no updating of dietary data over time.

In conclusion, the long-term epidemiological studies on the risk of developing T2D show heterogeneous results, but the most robust studies do not report any effects.

### Other effects

#### Effects on lipid parameters

Of the 20 randomised controlled experimental studies analysed, the majority focused on aspartame or stevia extracts. Compared to a placebo, aspartame consumption had no effects on triglycerides or cholesterol concentrations (either total, HDL, LDL or VLDL cholesterol) in various populations (healthy, T2D and overweight subjects) for periods ranging from 13 to 28 weeks. Compared to a caloric sweetener (sucrose, glucose or fructose), of the five identified studies, two showed a modest significant improvement in lipid profile (TG and/or total cholesterol) in the group that received aspartame, still with no differences compared to the placebo. Three studies, two in T2D patients, assessed the effect of stevia extracts on lipid parameters and showed no differences compared to a placebo. Studies using other types of IS (cyclamate, sucralose, IS mixture or unspecified IS) also showed no effects on the assessed lipid parameters. Of the four identified cohort studies, most showed no effects on lipid parameters related to the consumption of artificially sweetened beverages. A single study reported a positive association between the consumption of these beverages and an increase in TG concentrations associated with a lowering of HDLc [67].

In conclusion, the majority of observational studies showed no effects on lipid profile related to IS. Two studies reported that replacing sugars with aspartame reduced plasma triglyceride concentrations but the data are too limited to conclude that IS have a beneficial effect on lipid profile.

#### Effects on pre-term deliveries

Two epidemiological studies are available. In the first [68], a dose-effect relationship was observed, which meant that the risk of pre-term delivery was higher in the heaviest consumers of artificially sweetened beverages (OR (95 % CI) 1.38 [1.15-1.65] for ≥ 1 serving of artificially sweetened carbonated soft drinks/d). In addition to this Danish study, another study, with a similar methodology and including over 60,000 pregnant women, suggested that the consumption of artificially sweetened beverages and sugar-sweetened beverages was associated with an increased risk of spontaneous or induced pre-term delivery (OR (95 % CI) 1.11 [1.00-1.24] for ≥ 1 serving of artificially sweetened soft drinks/d). However, although the association was stronger for sugar-sweetened beverages, the authors concluded that they could not determine whether this risk was caused by the effects of these beverages or by other associated dietary or socio-economic factors [69].

Based on the available data, it is not possible to identify any benefits or draw any conclusions regarding the risk related to the consumption of intense sweeteners during pregnancy, in terms of maternal health, obstetrical parameters or newborn health.

#### Effects on cancer

The relationship between IS consumption and cancer in humans was assessed in 55 scientific studies. Thirty-nine of these studies involved the urinary tract and 32 focused exclusively on bladder cancer. The other studies assessed the relationship between IS consumption and the risk of brain cancer (four studies), digestive system cancer (six studies) or other cancers (five studies). Except in the studies focusing on bladder cancer, the IS in question were not identified by the authors. The relationship between saccharine consumption and bladder cancer was the most commonly studied, given that data were available in rodents [70]. The results of studies in humans are conflicting. Based on the analysis of data in humans, it is not possible to determine a relationship (whether for saccharine or for the other studied IS), since the studies did not adjust their results for major confounding factors such as exposure to chemical pollutants. Regarding kidney, brain, digestive system and breast cancers, the data are more limited and do not show any relationship with IS consumption. A recent cohort study examining the risk of lymphoma and leukaemia suggested an increased risk of non-Hodgkin lymphomas and multiple myelomas in males consuming more than one serving (355 mL) per day of artificially sweetened beverages and in the heaviest consumers of aspartame (as a table-top sweetener and in beverages) compared to non-consumers [71]. No significant association was reported in women. The authors specified that due to the differences in the results by sex, the results should be interpreted with caution. Moreover, this study did not take into account exposure to chemical pollutants as a confounding factor. However, it is worth noting that this study attempted to take into account, in its statistical analysis, changes in the individual consumption of artificial sweeteners over time, although little information is available regarding the methodology.

On the whole, the epidemiological studies do not show any effects of IS consumption on cancer risk. Only one recent study suggested a relationship between the consumption of beverages containing IS and the occurrence of non-Hodgkin lymphomas and myelomas, and additional studies are required.

#### Neurological effects

Regarding the potential neurological effects of IS, only aspartame has been studied. There are two studies in healthy adults [72, 73]. No effects of aspartame on the measured parameters (reaction time, headaches, hunger, sedation, electroencephalographic parameters) were observed. The study undertaken in epileptic subjects [74] showed no statistically significant difference between aspartame and the placebo on the incidence of epileptic seizures. The four available studies on migraine subjects [75–78] show conflicting results. However, no conclusion can be drawn due to their poor methodological quality (no adjustment) and the subjective nature of the measured effects (using non-validated self-questionnaires). Regarding children, there are two studies, one in epileptic children [79] and the other in hyperactive children [80], showing no significant effects of aspartame.

Some studies with significant methodological limitations suggested that aspartame consumption may be involved in triggering epileptic seizures and migraines but no conclusions can be drawn regarding the occurrence of such a risk from the data as a whole.

## Discussion and recommendations

The use and consumption of intense sweeteners have risen sharply over the last twenty years, probably due to concerns linked to the doubling of prevalence of overweight and obesity. While the potential risks of each intense sweetener are assessed before their authorisation, no general assessment of the overall nutritional risks and benefits of these products has been conducted at the European level up to now [81–83].

After an analysis of all of the scientific literature, it appears that, despite a large number of studies, the data are insufficient to determine any long-term nutritional benefits related to the consumption of products containing IS as sugar substitutes. The available data do not show any risks related to IS consumption. However, due to the limited number of studies, it is not possible to rule out potential long-term risks related to IS consumption in specific populations, particularly adult daily consumers and children. It is important to note that before their authorization to be on the market, the potential risks of each intense sweetener are assessed and an acceptable Daily Intake (ADI) is set for each IS. Based on several food surveys, an estimate of the dietary intakes of the ISs currently used in various countries shows that in all study populations (i.e. adults and children over the age of three, pregnant women and young diabetics) and irrespective of the IS taken into consideration, the mean and 95^th^ percentile intakes are lower than the ADIs.

The use of intense sweeteners as a substitute for sugar in most cases engenders a short term reduction in caloric intake due to the low calorie levels of these substances and the lack of compensation. However, the available data cover insufficient time periods for guaranteeing that this effect is maintained in the long term. Moreover, studies of weight control in adults and children have reported conflicting associations. Certain observational studies show that intense sweetener use is paradoxically associated with weight gain, although the causality of this relationship has not been established.

The consumption of intense sweeteners was not shown to have any beneficial effects on prevention of type 2 diabetes; similarly, their regular consumption as a sugar substitute does not appear to have any beneficial effect on regulating blood glucose concentrations. For the risks of developing cancer, type 2 diabetes, or premature births, the data available to date do not enable a link to be established between onset of these risks and the consumption of intense sweeteners. A few studies do however highlight the need to obtain further knowledge on the link between intense sweeteners and certain risks and in specific populations, particularly adults daily consumers and children.

This review of the scientific literature revealed some gaps that should be filled and areas of research that should be explored. There were differences between the results of randomised controlled trials and the results of observational studies. Furthermore, the studies assessing the effects of replacing sugars with IS on eating behaviour or energy intake were undertaken over the short term and should be supplemented with long-term studies. Two types of additional studies seem necessary to clear up these differences. On the one hand, longer-term (at least one year) blind, placebo-controlled intervention studies would shed further light on the metabolic and physiological effects of IS. On the other hand, intervention studies in which IS are consciously consumed would help to understand potential changes in eating behaviour related to the replacement of sugars with IS in near-real-life conditions. There are also very few data on the long-term impact of IS on food preferences. Therefore, it appears necessary to study the effects of IS consumption on dietary choices. In most of the cohort studies, the consumption of artificially sweetened beverages was reported only when the subjects were first included, and subsequent consumption was not taken into account. These studies assessed only the consumption of artificially sweetened beverages, and not the total consumption of artificial sweeteners. It is also difficult to distinguish between the effects of the various IS consumed alone and their effects when combined with other IS. Future cohort studies should be capable of taking into account qualitative and quantitative changes in the consumption of artificially sweetened products and adapted dietary questionnaires, to accurately and specifically assess IS consumption. Specific populations such as pregnant women, children, diabetic subjects and regular IS consumers have not been adequately studied. It appears necessary to further study the effects of IS in these populations. Likewise, it appears necessary to determine the repercussions of IS consumption during the peri-natal phase on offspring (under the 'foetal programming' assumption). Some data suggested that the potential effects of IS on changes in weight or the incidence of diabetes may vary depending on initial corpulence. Due to the increasing prevalence of obesity, it appears necessary to study potential interactions between corpulence and IS consumption in terms of the risk of weight gain or diabetes. At last, the literature on steviol glycosides is still sparse and should be enhanced, particularly given the recent growth in their use in beverages and foods.

## Conclusions

No beneficial effects have been shown that provide grounds to recommend regular IS consumption for adults or children. Moreover, the available data do not show the occurrence of risk in occasional consumers. However, based on the epidemiological data currently available, it is not possible to completely rule out certain risks in the event of regular, prolonged consumption. Therefore, for the general population, the overall assessment of potential risks and benefits does not justify the long-term use of IS as sugar substitutes, particularly in beverages, which are their main vector. Lastly, in a nutritional policy context in which one of the main objectives is the reduction of sugar intake in the general public, as pointed out by the 2015 WHO guidelines on sugar intake, this review points out that no meaningful data exist that justify encouraging the substitution of sugars by intense sweeteners. This objective of reduction of sugar intake levels should be reached through a reduction in sweet tasting foods in general at an early age. It should therefore be recommended that artificially-sweetened and sugar-sweetened soft drinks shall not be consumed as a replacement for water.
