# Erratum to: Review of the nutritional benefits and risks related to intense sweeteners

**DOI:** 10.1186/s13690-015-0102-z

**Published:** 2015-10-23

**Authors:** Olivier Bruyère, Serge H. Ahmed, Catherine Atlan, Jacques Belegaud, Murielle Bortolotti, Marie-Chantal Canivenc-Lavier, Sybil Charrière, Jean-Philippe Girardet, Sabine Houdart, Esther Kalonji, Perrine Nadaud, Fabienne Rajas, Gérard Slama, Irène Margaritis

**Affiliations:** Department of Public Health, Epidemiology and Health Economics, University of Liege, CHU Sart Tilman, Bât B23, 4000 Liège, Belgium; CNRS UMR 5293/Université de Bordeaux, Bordeaux, France; Centre Hospitalier de Luxembourg, Luxembourg, Luxembourg; Université de Picardie, Amiens, France; Centre Hospitalier Universitaire Vaudois, Lausanne, Suisse Switzerland; Centre des Sciences du Goût et de l’Alimentation - INRA Dijon, Dijon, France; Université Claude Bernard Lyon 1, Hospices Civils de Lyon, Inserm U1060, Lyon, France; Sorbonne Universités, UPMC Univ Paris 06, Paris, France; French Agency for Food, Environmental and Occupational Health & Safety (Anses), Maisons-Alfort, France; INSERM 855/Université Claude Bernard Lyon 1, Lyon, France; Hôtel-Dieu Hospital, René Descartes University-Paris V, Paris, France

Unfortunately, the original version of this article [1] contained an error.

The author’s names were included incorrectly, the surnames were presented before the forename:

Bruyère Olivier, Ahmed H. Serge, Atlan Catherine, Belegaud Jacques, Bortolotti Murielle, Canivenc-Lavier Marie-Chantal, Charrière Sybil, Girardet Jean-Philippe, Houdart Sabine, Kalonji Esther, Nadaud Perrine, Rajas Fabienne, Slama Gérard and Margaritis Irène

The author list has been corrected in the original article and is also included correctly below:

Olivier Bruyère, Serge H. Ahmed, Catherine Atlan, Jacques Belegaud, Murielle Bortolotti, Marie-Chantal Canivenc-Lavier, Sybil Charrière, Jean-Philippe Girardet, Sabine Houdart, Esther Kalonji, Perrine Nadaud, Fabienne Rajas, Gérard Slama, Irène Margaritis
